# Controlling Horizontal Cell-Mediated Lateral Inhibition in Transgenic Zebrafish Retina with Chemogenetic Tools

**DOI:** 10.1523/ENEURO.0022-20.2020

**Published:** 2020-10-22

**Authors:** Billie Beckwith-Cohen, Lars C. Holzhausen, Scott Nawy, Richard H. Kramer

**Affiliations:** 1 Vision Science Graduate Program, University of California, Berkeley, School of Optometry, Berkeley, CA 94720; 2 Department of Molecular and Cell Biology, University of California, Berkeley, Berkeley, CA 94720

**Keywords:** feedback inhibition, fluorescent protein, horizontal cell, lateral inhibition, photoreceptor, retina

## Abstract

Horizontal cells (HCs) form reciprocal synapses with rod and cone photoreceptors, an arrangement that underlies lateral inhibition in the retina. HCs send negative and positive feedback signals to photoreceptors, but how HCs initiate these signals remains unclear. Unfortunately, because HCs have no unique neurotransmitter receptors, there are no pharmacological treatments for perturbing membrane potential specifically in HCs. Here we use transgenic zebrafish whose HCs express alien receptors, enabling cell-type-specific control by cognate alien agonists. To depolarize HCs, we used the Phe-Met-Arg-Phe-amide (FMRFamide)-gated Na^+^ channel (FaNaC) activated by the invertebrate neuropeptide FMRFamide. To hyperpolarize HCs we used a pharmacologically selective actuator module (PSAM)-glycine receptor (GlyR), an engineered Cl^–^ selective channel activated by a synthetic agonist. Expression of FaNaC or PSAM-GlyR was restricted to HCs with the cell-type selective promoter for connexin-55.5. We assessed HC-feedback control of photoreceptor synapses in three ways. First, we measured presynaptic exocytosis from photoreceptor terminals using the fluorescent dye FM1-43. Second, we measured the electroretinogram (ERG) b-wave, a signal generated by postsynaptic responses. Third, we used Ca^2+^ imaging in retinal ganglion cells (RGCs) expressing the Ca^2+^ indicator GCaMP6. Addition of FMRFamide significantly decreased FM1-43 destaining in darkness, whereas the addition of PSAM-GlyR significantly increased it. However, both agonists decreased the light-elicited ERG b-wave and eliminated surround inhibition of the Ca^2+^ response of RGCs. Taken together, our findings show that chemogenetic tools can selectively manipulate negative feedback from HCs, providing a platform for understanding its mechanism and helping to elucidate its functional roles in visual information processing at a succession of downstream stages.

## Significance Statement

Horizontal cells (HCs) are laterally projecting interneurons that share reciprocal synaptic connections with photoreceptors, an arrangement that establishes the antagonistic center/surround receptive field properties of downstream neurons in the retina and onward into the brain. HC-mediated lateral inhibition was discovered over half a century ago, yet its underlying synaptic mechanisms remain incompletely understood. This is largely because the reciprocal synapse complicates selective manipulation of HCs alone. Here, we use chemogenetic tools to bypass photoreceptors and directly manipulate HC membrane potential to reveal feedback effects on sequential steps in synaptic processing of visual information.

## Introduction

Lateral inhibition in the vertebrate retina depends on reciprocal synaptic communication between rod or cone photoreceptors and horizontal cells (HCs; Thoreson and Mangel, 2012; Kramer and Davenport, 2015). Photoreceptors have a sign-preserving excitatory synapse onto HCs, and HCs exert wide-field sign-inverting negative feedback and narrow-field positive feedback back onto the photoreceptors (Baylor et al., 1971; Jackman et al., 2011). The net effect of HC feedback is that bipolar cells, which carry the light response from the outer to the inner retina, exhibit an antagonistic center-surround receptive field, a property critical for enhancing contrast sensitivity in the visual system and enabling high-acuity vision. In cold-blooded vertebrates, HC feedback also plays critical roles in light adaptation and color information processing (Thoreson and Mangel, 2012). Despite decades of study, the mechanisms underlying HC feedback onto photoreceptors are still being unraveled, largely because the reciprocal connection makes it difficult to restrict experimental manipulation to one specific side of the synapse or the other. Light can uniquely elicit feed-forward synaptic signals from photoreceptors, but eliciting a feedback signal specifically from the HCs has required intracellular manipulation of membrane potential with sharp electrodes or larger patch clamp pipettes. Practically speaking, these recordings can only be implemented in one or two cells at a time.

Optogenetics and chemogenetics have emerged as popular methods for perturbing membrane potential in entire populations of genetically-targeted cells without requiring insertion of electrodes (Atasoy and Sternson, 2018). While the intrinsic light-sensitivity of photoreceptors complicates the use of optogenetics in the retina, chemogenetics is well suited for manipulating specific cell types without the use of light (Drinnenberg et al., 2018). Chemogenetics involves the exogenous expression of a receptor that is foreign to an organism’s nervous system, with activation of the receptor brought about by an agonist that is also foreign and selective.

One such receptor is Phe-Met-Arg-Phe-amide (FMRFamide)-activated Na^+^ channel (FaNaC), a rare example of a neuropeptide receptor that is not metabotropic, but an ionotropic (Na^+^-selective) ion channel. FaNaC, which is normally expressed in mollusks but not in vertebrates (Golubovic et al., 2007), is activated by the neuropeptide FMRFamide, which is also absent from the vertebrate nervous system. In the mammalian brain, application of FMRFamide onto neurons genetically targeted to express FaNaC leads to depolarization and stimulation of action potential firing (Schanuel et al., 2008). Pharmacologically selective actuator modules (PSAMs) are another type of foreign receptor. PSAMs were engineered specifically to operate orthogonally to natural chemical neurotransmission (Magnus et al., 2011). The PSAM used here was derived from a chimeric combination between the ligand-binding domain of a nicotinic acetylcholine receptor and the transmembrane domain of a Cl^–^ conducting glycine receptor (GlyR; PSAM-GlyR), yielding an engineered ligand-gated Cl^–^ channel.

Transgenic zebrafish models are well suited for exploring neural function, especially with respect to vision. Zebrafish have a short generation time, breed in large numbers, and are easy to maintain in laboratories. Molecular strategies developed over decades enable efficient generation of transgenic lines (Niklaus and Neuhauss, 2017). The transparency of zebrafish embryos enables functional *in vivo* imaging of the retina, brain, and spinal cord in completely intact animals (Dreosti et al., 2009). Zebrafish larvae can also be used for high throughput behavioral screens, taking advantage of their permeability to many small molecule drugs directly from the water in which they swim (Cassar et al., 2017).

Previous studies on zebrafish supported the hypothesis that a change in extracellular pH accounts for most of the negative feedback signal from HCs onto cones that underlies lateral inhibition (Wang et al., 2014; Beckwith-Cohen et al., 2019). An as yet unidentified signal mediates positive feedback from HCs onto cones (Jackman et al., 2011), a process that amplifies lateral inhibition. Here, we use FaNaC and PSAM-GlyR in transgenic zebrafish, targeted with cell-type-specific promoters, to leap-frog over the photoreceptors and directly manipulate HC membrane potential. We examine the effects of HC manipulation at progressive downstream stages in retinal signaling, first at the level of synaptic exocytosis from cone photoreceptors, second at the level of postsynaptic responses of bipolar cells, and finally at the level of center versus surround responses in retinal ganglion cells (RGCs).

## Materials and Methods

All animal procedures were performed in accordance with the University of California, Berkeley Animal Care and Use Committee regulations and were conducted in adherence to the Association for Research in Vision and Ophthalmology (ARVO) Statement for the Use of Animals in Ophthalmic and Vision Research as well as the Society for Neuroscience Policies on the Use of Animals and Humans in Neuroscience Research.

### Animal husbandry

Adult or larval zebrafish (*Danio rerio*) of either sex were used in all experiments. Animals were housed in a recirculating water system at a density of five to 10 adult fish per 1 l of water. Animals were reared at 27–28.5°C in mixed-sex groups of the same date of birth on a 14/10 h light/dark cycle. The Zeitgeber time of light onset at the animal facility was 9 A.M., and light offset was 11 P.M. Fish were bred no more than once per week. Water quality was checked twice daily for pH, temperature, and conductivity, and once weekly for nitrite, nitrate, alkalinity, and general hardness.

### DNA constructs and transgenic fish

Transgenic zebrafish expressing FaNaC were generated as described elsewhere (Wang et al., 2014; ZFIN catalog #ZDB-ALT-140924–3, RRID:ZFIN_ZDB-ALT-140924-3) and were activated using its cognate agonist FMRFamide (Sigma-Aldrich, P4898; CAS number 64190-70-1). PSAM-expressing fish were generated specifically for this study (ZFIN catalog #ZDB-ALT-181031-3, RRID:ZFIN_ZDB-ALT-181031-3). The PSAML141F,Y115F:GlyR (RRID:Addgene_32480, Magnus et al., 2011) was subcloned into pDONR221 to obtain pME-PSAM-GlyR. For bicistronic expression of PSAM-GlyR with the green fluorescent protein eGFP, the coding sequence of the internal ribosome entry site (IRES) viral peptide was inserted at the 5′ end of the eGFP coding sequence in p3E-eGFP to produce p3E-IRES:eGFP. To generate the PSAM constructs, we recombined p5E-MCS Cx55.5 (Shields et al., 2007; kindly provided by Maarten Kamermans, Netherlands Institute for Neuroscience), pME-PSAM AA0416 Y115F-L141F-GlyR, p3E- IRES:eGFP, and pDestTol2CG2 from the Tol2kit#395 (RRID:Addgene_63156) for making transgenic fish. One-cell-stage or two-cell-stage zebrafish (*D. rerio*, AB strain) embryos were microinjected with the DNA constructs together with Tol2 mRNA for a higher germline transmission rate (Kwan et al., 2007). The transgene-positive F0 founders were selected by screening for green heart fluorescence (myl7:GFP, aka cmlc2) in embryos at 2–4 d postfertilization (dpf; Kwan et al., 2007) and raised at 28.5°C in a 14/10 h light/dark cycle. Adult F0 fish were inbred, and their transgene-positive progeny were screened by green heart fluorescence, raised, and used for imaging experiments and immunohistochemistry. The PSAM-GlyR is activated by its cognate agonist PSEM^89S^ (Tocris Biosciences; IUPAC name: N-(3S)−1-Azabicyclo[2.2.2]oct-3-yl-2,5-dimethoxybenzamide trifluoroacetate, CAS number: 1336913-03-1). Transgenic zebrafish expressing GCaMP6f under the pan-neuronal promoter HuC [Tg2(elavl3:GCaMP6f), RRID:ZFIN_ZDB-GENO-180220-2, ZFIN catalog #ZDB-FISH-180220-12] were kindly provided by the Florian Engert Lab. To generate HuC:GCaMP and FaNaC double transgenic fish or HuC:GCaMP and PSAM-GlyR double transgenic fish, the HuC:GCaMP transgenic fish line was crossed with either the FaNaC or PSAM-glyR fish line. Double transgenic expression was confirmed by screening embryos at 3 dpf for concurrent expression of a green fluorescent heart, and a green fluorescent nervous system (i.e., brain and spinal cord).

### Tissue preparation

Fish were dark adapted for at least 30 min before dissection. Adults were killed by immersion in an overdose concentration of 400 mg/l MS-222, with decapitation as a secondary means of euthanasia. Zebrafish larvae <8 dpf used for electroretinogram (ERG) recordings were killed by decapitation followed by pithing, and recordings were performed on isolated eyes. For experiments performed on isolated retinas, dark-adapted retinas were dissected and isolated from the enucleated eyes in the dark, under infrared light, using IR viewers connected to the dissection microscope. The retinal pigment epithelium was manually separated from the neurosensory retina. Retinas were maintained in darkness in bubbled bicarbonate-buffered Ringer's solution. For flat-mount preparations, retinas were mounted onto a Biopore membrane (Millipore) with photoreceptors facing the membrane (Koizumi et al., 2007).

For gramicidin-perforated recordings from isolated HCs, adult zebrafish retinas were dissected and prepared for HC dissociation as previously described (Nelson et al., 2008). Briefly, retinas were incubated for 1 h at room temperature with gentle agitation in 1 ml of 0 Ca^2+^ saline (120 mm NaCl, 10 mm glucose, 2.5 mm KCl, 1.2 mm MgSO_4_, buffered to pH 7.7 with 3 mm HEPES), containing 30 units of papain (Worthington Biochemical) activated with 1 mm cysteine, and 2 mg/ml hyaluronidase (Worthington Biochemical). Retinas were washed with Ca^2+^ (2.2 mm) saline solution containing 1% BSA to inactivate papain, and then gently triturated 3 times in saline solution and plated onto plastic-coated 35-mm dishes, which served as the recording chamber.

### Patch clamp recording

To measure responses to FMRFamide, PSEM^89S^, GABA, and muscimol, HCs were recorded with the perforated patch configuration using gramicidin as the perforating agent. The bathing solution contained the following: 120 mm NaCl, 2.5 mm KCl, 1.2 mm MgSO_4_, 2.2 mm CaCl_2_, and 3.0 mm HEPES, pH 7.7. A stock solution of gramicidin in ethanol (5 mg/ml) was prepared and diluted into the pipet solution (130 mm KCl, 3 mm NaCl, and 10 mm HEPES, pH 7.4) at a ratio of 6 μl/ml. Pipets were pulled to a resistance of ∼10–12 MΩ using a vertical puller (PC-100, Narishige Scientific Instruments). Following seal formation, the presence of a membrane potential in the current clamp recording configuration confirmed successful perforation, usually within the first minute. PSEM^89S^ (500 μm), GABA (1 mm), and muscimol (100 μm) were applied with 100-ms pulses of positive pressure (1–2 psi) from a second pipet positioned ∼100 μm from the cell. FMRFamide (30 μm) was bath applied for 5 min before measuring changes in membrane potential. FMRFamide was purchased from Sigma-Aldrich. Other agonists were purchased from Tocris Biosciences. Recordings were made using the MultiClamp 700b patch clamp amplifier, and pClamp software (Molecular Devices). All reported values were corrected for junction potentials, calculated using pClamp software.

### FM1-43 imaging experiments

We used the fluorescent amphipathic dye FM1-43 to quantify synaptic exocytosis from cone photoreceptor terminals, as described previously (Rea et al., 2004; Choi et al., 2005). First, the isolated retina was treated with FM1-43 (30 μm) in normal saline containing the following: 100 mm NaCl, 2.5 mm KCl, 1 mm MgCl_2_, 1 mm CaCl_2_, 0.4 mm ascorbic acid, 20 mm dextrose, and 25 mm NaHCO_3_ bubbled with 5% CO_2_ and 95% O_2_ at 21°C for 15 min in darkness. This allows dye to accumulate in recycling synaptic vesicles. The loading period was followed by a 15-min wash with 0 Ca^2+^, 1 mm EGTA saline to suppress premature unloading of dye by Ca^2+^-dependent exocytosis. Residual dye trapped in the surface membrane was removed with Advasep-7 (1 mm; Kay et al., 1999). After the wash period, Ca^2+^-free saline was replaced with normal Ca^2+^-containing saline to allow Ca^2+^-dependent exocytosis of dye-loaded vesicles, leading to loss of FM1-43 fluorescence. Dye loss was visualized with two-photon microscopy, and the decrease in fluorescence intensity over time was normalized to the initial fluorescence, before adding back Ca^2+^. At the end of each experiment, high K^+^ saline (50 mm KCl, iso-osmotically replacing NaCl) was applied for 15 min to elicit exocytosis of all releasable vesicles. Any remaining background fluorescence, attributable to dye trapped in unreleasable compartments, was normalized to zero.

Two-photon imaging was conducted with a Zeiss 510 microscope equipped with a MaiTai (Spectra Physics) mode-locked Ti:sapphire laser (860 nm) and a 40× achroplan, 0.8 NA water-immersion objective. Images were acquired at a frame rate of 10 Hz with Zeiss LSM software and analyzed with Scion Image software. Continuous imaging of FM1-43 resulted in some degree of steady state light adaptation (Choi et al., 2005). Optical sections focused 300 nm apart were obtained to image throughout the thickness of the outer plexiform layer (OPL). Z-stacks encompassing the entire OPL were corrected for drift and summed with FiJi ImageJ to generate average intensity z-projections.

### ERG recordings

Corneal ERGs were recorded from dark-adapted eyes isolated from <8 dpf zebrafish larvae in oxygenated Ringer’s solution. Animals were dark adapted for at least 12 h before experiments. ERGs were recorded from isolated eyes on filter paper by using a glass pipette (G150TF-4, Warner Instruments) pulled to a diameter of 10–12 μm (Flaming/Brown type micropipette puller, Sutter Instruments, P-97) placed onto the central cornea with a hydraulic micromanipulator. Each pipette tip was individually examined and smoothed as needed (MF-83 microforge, Narishige Scientific Instruments). A platinum wire placed beneath the moist filter paper was used as a reference electrode. Monochromatic light was presented to eyes via a monochromator with a 150-W Xenon high stability lamp (Polychrome V, Till Photonics GmbH). Three repetitive 2-s isoluminant pulses of 400-nm light with 15-s interstimulus intervals were used to elicit ERG responses and were averaged for each of eight measured time points. FMRFamide or PSEM^89S^ was added at *t* = 0 min. The b-wave amplitude was measured by subtracting the baseline value or the minimal value of the first negative peak to the maximal value of the first positive peak (Evers and Gouras, 1986). For comparisons between treatment groups, the ERG was normalized to the b-wave peak amplitude before drug application. Data were acquired and processed with a custom-written MATLAB routine. Wild-type (WT) AB-strain zebrafish or transgene-negative larvae of the same spawning were used for control experiments.

### GCL calcium imaging

For two-photon Ca^2+^-imaging experiments, we used a transgenic zebrafish expressing the genetically encoded calcium indicator GCaMP6f, under the control of a pan-neuronal promoter (HuC). Whole retinas were isolated from dark-adapted adult zebrafish and flat-mounted on Biopore membrane with the RGC side facing the top of the chamber. Retinas were continuously perfused with bicarbonate-buffered solution containing the following: 100 mm NaCl, 2.5 mm KCl, 1 mm MgCl_2_, 1 mm CaCl_2_, 0.4 mm ascorbic acid, 20 mm dextrose, and 25 mm NaHCO_3_ bubbled with 5% CO_2_ and 95% O_2_. We focused on a single image plane in the ganglion cell layer and used ImageJ (https://rsbweb.nih.gov/ij) to define regions of interest, corresponding to individual somata. We used 920-nm light for GCaMP6f two-photon excitation. Emitted light was passed through a 530-nm barrier filter.

### Immunohistochemistry

Transgenic and WT zebrafish of either sex were dark adapted, killed as previously detailed, and their eyes were enucleated. For tissue fixation 4% paraformaldehyde was prepared from 100% paraformaldehyde diluted in 1× PBS buffered to a pH of 7.4. The eyes were fixed for at least 1 h. After fixation the eyes were rinsed in PBS, and cryoprotected by immersion for 1 h in 15% sucrose followed by 1 h in 30% sucrose solutions. Eyes were sectioned at a thickness of 14 μm using a microtome (ThermoFisher Scientific Microm HM550) and kept in a −20°C freezer until immunohistochemistry was performed.

Immunohistochemistry using an antibody to GFP was performed to enhance detection of labeling in PSAM-GlyR fish. Details have been reported elsewhere (Beckwith-Cohen et al., 2019). Briefly, sections were incubated for 30 min at 4°C in blocking solution consisting of 1% Triton X-100 (Sigma-Aldrich), 1% bovine serum albumin fraction V (MP Biomedicals LLC, 160069) at 4°C. Sections were rinsed in PBS and then incubated with mouse anti-GFP monoclonal unconjugated antibody (ABCAM ab1218; RRID:AB_298911) at a 1:400 dilution and blocking solution for 2 h. After washing, sections were immersed in 4°C blocking solution including Alexa Fluor 488 goat anti-mouse igG (ThermoFisher Scientific, catalog #A-11001; RRID:AB_2534069) at a 1:1000 dilution with gentle agitation for 1 h at 100 rpm. Sections were then rinsed again for three cycles in chilled PBS, air dried and sealed using Fluoromount-G with DAPI mounting solution (Invitrogen). Negative controls included sections of the same tissue without the primary antibody. Sections were imaged using a confocal microscope (Carl Zeiss LSM880). GFP and mCherry were excited at 488 and 647 nm using Argon and HeNe lasers respectively; DAPI was excited using ultraviolet light at 348 nm. Live larval images were obtained from 1 to 3 dpf zebrafish larva embedded in agarose gel as previously described (Williams et al., 2013). Larvae were imaged using a confocal microscope (Carl Zeiss LSM880).

### Statistical analysis

Statistical significance for sample numbers less than ten was determined by using the two-sided nonparametric Mann–Whitney *U* test. Significance for multiple group comparisons was determined using one-way ANOVA followed by the two-tailed Student’s *t* test. Significance was otherwise determined by two-tailed Student’s *t* test or rank sum test as indicated. Effects were considered significant at *p* < 0.05. Data points and error bars represent the mean ± SEM. Statistics were performed using Microsoft Office Professional Excel version 2016 or MATLAB 9.

## Results

### Chemogenetic tools for manipulating HCs

**Figure 1.**
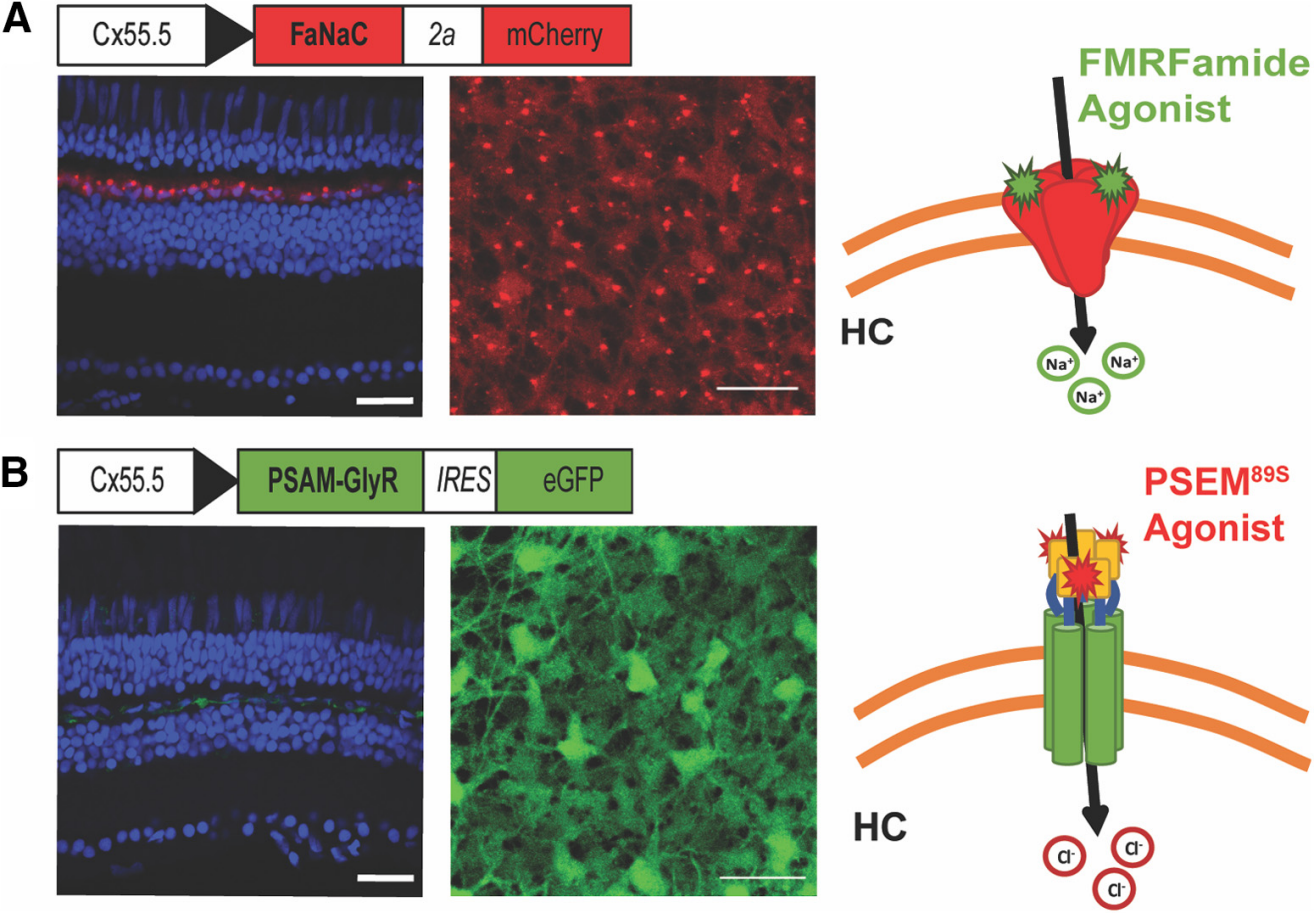
Strategy for chemogenetic manipulation of HC membrane potential. ***A***, A bicistronic construct containing the molluscan FaNaC from *H. aspersa* and the red fluorescent protein mCherry, both under the control of the Cx55.5 promoter, were transgenically expressed in HCs of zebrafish causing red fluorescence specific to the HC layer. mCherry exhibits intrinsic fluorescence seen in confocal imaging of a fixed retinal section (left) and in two-photon imaging of a fresh retinal flat mount (right). Puncta with saturating expression of mCherry likely represent protein aggregates within the Golgi apparatus of HCs. A diagram illustrates how binding of the agonist FMRFamide causes Na^+^ influx thereby depolarizing the membrane potential. ***B***, A bicistronic construct containing the PSAM-GlyR and the green fluorescent protein eGFP, both under the control of the Cx55.5 promoter, were transgenically expressed in HCs of zebrafish causing green fluorescence specific to the HC layer. eGFP was immunolabeled with Alexa Fluor 488 for confocal imaging of a fixed retinal section (left) and its intrinsic fluorescence is seen in two-photon imaging of a fresh retinal flat mount (right). A diagram illustrates how binding of the designer agonist PSEM^89S^ causes Cl^–^ influx thereby clamping the membrane potential to ECl. Nuclei are stained with DAPI (blue). Scale bar: 20 μm.

To use a chemogenetic approach to manipulate HC membrane potential, we used two zebrafish lines. One was a previously developed transgenic line (Wang et al., 2014) that expresses the FaNaC from the snail *Helix aspersa*. FaNaC expression was controlled by the connexin 55.5 promoter, which in the retina, enables specific expression in HCs (Shields et al., 2007). The transgenic insert also encoded the fluorescent protein mCherry, which allowed us to verify that the only retinal cells expressing FaNaC were HCs (Fig. 1*A*). We developed a second transgenic zebrafish line that expresses the PSAM-GlyR (Magnus et al., 2011). PSAM-GlyR was also expressed under the control of the connexin 55.5 promoter, along with eGFP to allow visualization of cells expressing the alien channel (Fig. 1*B*). Because PSAM-GlyR is a Cl^–^ channel, the effect of PSEM^89S^ on membrane potential is dependent on the value of ECl, which varies depending on the concentrations of Cl^–^ in the bathing solution and in the patch pipette. Binding of the designer agonist PSEM^89S^ to PSAM-GlyR should open Cl^–^ channels and drive the membrane potential toward ECl.

**Figure 2.**
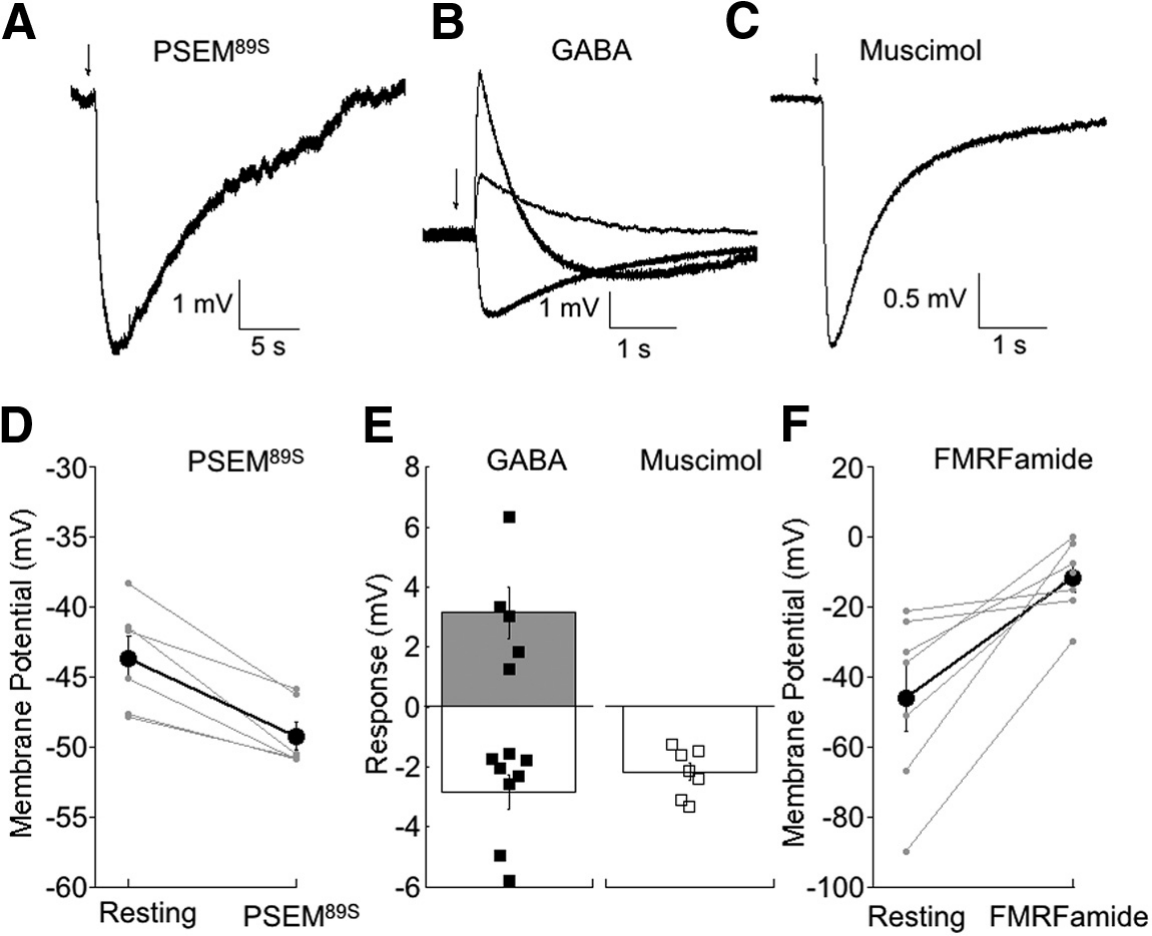
Monitoring changes in membrane potential elicited by FMRFamide and PSEM^89S^. ***A***, Voltage response to a 100-ms puff of PSEM^89S^ (500 μm) in an isolated HC recorded with the gramicidin perforated patch configuration. A puff was delivered at the time indicated by the arrow. Although the concentration of PSEM^89S^ was 500 μm in the pipette, the concentration of the drug is estimated to be diluted ∼50-fold at the cell (Firestein and Werblin, 1989), positioned ∼100 μm from the puffer pipette. ***B***, Traces from three different HCs showing responses to 100-ms puffs of GABA (1 mm), chosen to highlight the variability of responses to GABA that were observed between cells. ***C***, Response to 100-ms application of muscimol (100 μm), a GABA_A_ receptor agonist. Responses were uniformly hyperpolarizing in all cells tested. ***D***, Summary data showing the resting membrane potential, and the membrane potential at the peak of the response to PSEM^89S^. Large bolded symbols are the mean ± SEM for each condition (*n* = 6). ***E***, Summary of the change in membrane potential evoked by puffs of GABA or muscimol. Small closed symbols are the responses of individual cells to GABA (*n* = 11). Open symbols are the responses to muscimol (*n* = 7). Open boxes are the mean ± SEM for hyperpolarizing responses, and the shaded box is the mean ± SEM for depolarizing responses. The mean amplitude of the responses evoked by muscimol was not significantly different from the amplitude of hyperpolarization evoked by GABA (*p* = 0.53, rank-sum test). ***F***, Summary data for bath application of FMRFamide (30 μm). Large bolded symbols are the mean ± SEM for each condition (*n* = 7).

To determine how activation of the PSAM-GlyR receptor changes HC membrane potential, we used the perforated patch recording technique in which an ionophore added to the pipette solution inserts into the plasma membrane, providing access for measuring intracellular voltage. Gramicidin was chosen as the perforating agent as it forms cation channels that are impermeant to Cl^–^, thus preserving the normal internal Cl^–^ concentration (Kyrozis and Reichling, 1995). As our access resistance was typically ∼100 MΩ, we measured HC responses to agonists without attempting to manipulate membrane voltage with extrinsic current. A typical response to a 100-ms puff of PSEM^89S^ is shown in Figure 2*A*. PSEM^89S^ consistently hyperpolarized HCs, ranging from −3.0 to −9.1 mV (*n* = 6). Overall, PSEM^89S^ reversibly hyperpolarized HCs from a mean resting potential of −43.7 ± 1.6 mV to an average potential of 49.28 ± 1.0 mV (Fig. 2*D*). This finding is consistent with an ECl value more negative than the resting potential, resulting in hyperpolarization on activation of PSAM-GlyR. Application of PSEM^89S^ had no effect on cells from WT zebrafish (*n* = 10; data not shown).

Previous studies showed that the inhibitory neurotransmitter GABA can lead to depolarization of HCs (Miller and Dacheux, 1983; Djamgoz and Laming, 1987; Kamermans and Werblin, 1992; Grove et al., 2019). To better understand this process, we puffed GABA onto HCs and recorded membrane potential responses. Some HCs hyperpolarized −(2.6 ± 0.4 mV, *n* = 5), some depolarized (3.6 ± 0.8 mV, *n* = 4), and some exhibited a biphasic response (*n* = 2; Fig. 2*B*). The complex response suggests multiple conductance mechanisms activated by GABA. Fish HCs possess highly Cl^–^ selective GABAA receptors (Gilbertson et al., 1991; Kamermans and Werblin, 1992; Takahashi et al., 1995; Paik et al., 2003), but they also express GABA transporters (Malchow and Ripps, 1990; Cammack and Schwartz, 1993; Dong et al., 1994; Kreitzer et al., 2003; Nelson et al., 2008) which have a GABA-activated Na^+^-conducting pore (Krause and Schwarz, 2005). To test whether GABAA receptor activation is hyperpolarizing in HCs, we used muscimol, which is highly specific for GABAA receptors and has no effect on GABA transporters (Malchow and Ripps, 1990). We found that muscimol produced only hyperpolarizing responses (*n* = 7, range of −1.2 to −3.3 mV; Fig. 2*C*). Responses to GABA and muscimol are summarized in Figure 2*E*. Hence, the natural neurotransmitter GABA activates both a depolarizing transporter current and a hyperpolarizing GABAA current, with the net effect determined by the relative abundance or distribution of these targets. In contrast, the PSEM^89S^/PSAM-GlyR system exclusively hyperpolarizes HCs, making it a particularly effective tool for imparting inhibition.

Finally, we measured the effect of FMRFamide (30 μm) on HC membrane potential. HCs depolarized from −48.5 ± 8.1 mV (*n* = 7 cells) at rest to −11.6 ± 7.8 mV on application of FMRFamide, consistent with an increase in Na^+^ conductance (Fig. 2*F*). Thus, FMRFamide-mediated activation of FaNaC is an effective tool for depolarizing HCs.

### Chemogenetic manipulation of HCs alters FM1–43 destaining of cone terminals

To measure tonic exocytosis from photoreceptors, we used the synaptic vesicle dye FM1-43 (Betz and Bewick, 1992). FM1-43 is an amphipathic molecule that becomes fluorescent when it inserts itself into the plasma membrane. The dye is then washed off the tissue, with residual fluorescence attributable to dye trapped within vesicles internalized by endocytosis (Rea et al., 2004). Synaptic exocytosis releases the dye, causing a gradual decrease in tissue fluorescence. Photoreceptor terminals exhibit tonic exocytosis in darkness, resulting in progressive spontaneous release of dye.

**Figure 3.**
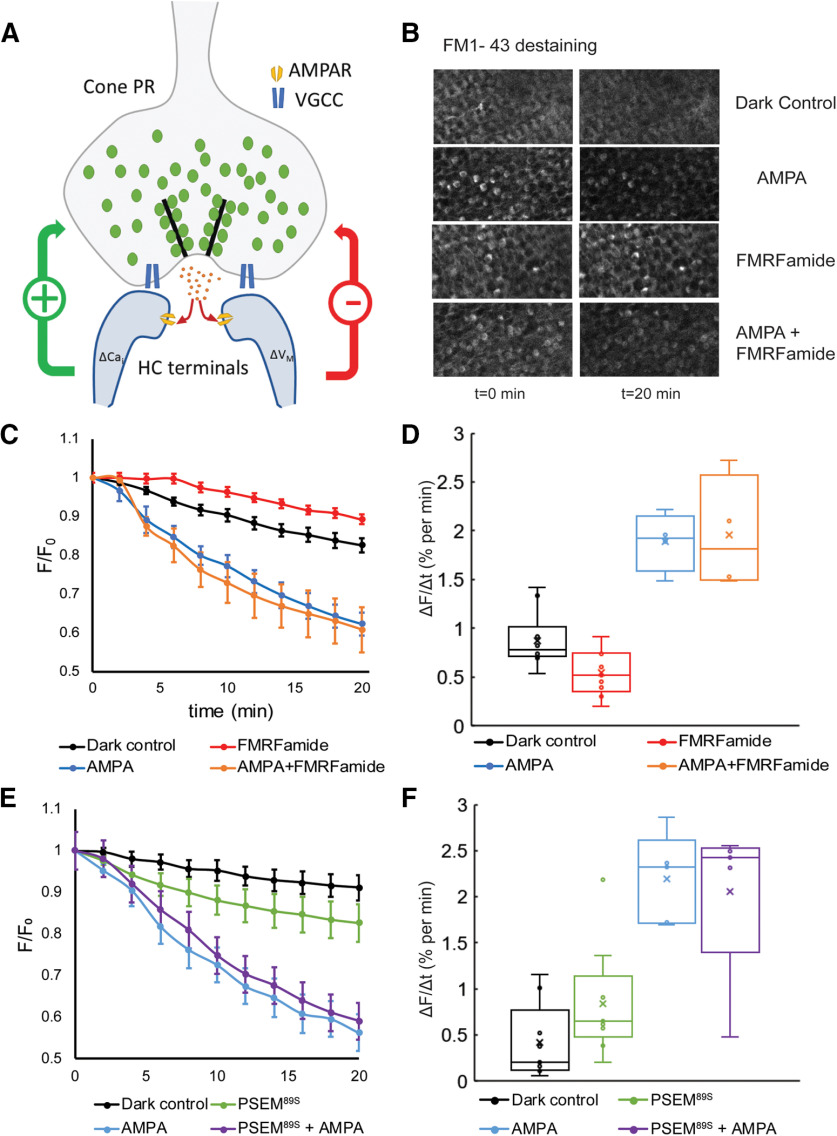
Chemogenetic activation of HCs alters vesicular release of FM1-43 from cone terminals. ***A***, Schematic representation of the mechanisms of negative (–) and positive (+) feedback of HCs onto cone photoreceptors (PR). VGCCs are voltage-gated Ca^2+^ channels, green circles are FM1-43-filled synaptic vesicles, orange dots are glutamate molecules in the synaptic cleft, AMPARs are AMPA receptors. Negative feedback is mediated by changes in the membrane potential of HCs, whereas positive feedback is mediated by increased intracellular Ca^2+^ in HCs, owing to influx of Ca^2+^ through Ca^2+^-permeant AMPA receptors. ΔCa_i_ is the change in intracellular Ca^2+^; ΔV_M_ is the change in HC membrane potential. ***B***, Two-photon scanned images of the photoreceptor terminal layer in FaNaC zebrafish retinas. Retinas were pretreated with FM1-43 to load recycling synaptic vesicles and then treated for 20 min with the indicated receptor agonist. AMPA (20 μm) increased the rate of dye loss (destaining) and FMRFamide (10 μm) decreased destaining, but the AMPA-elicited increase was dominant when the two agonists were applied together. ***C***, ***D***, Time course of FM1-43 destaining in FaNaC fish. Without added agonists, cone terminals released FM1-43 at 0.87 ± 0.08% per minute (*n* = 10 retinas). Adding AMPA accelerated destaining rate (1.89 ± 0.13%, *n* = 9 retinas, *p *<* *1 × 10−5). Adding FMRFamide (10 μm) decelerated destaining (0.54 ± 0.07% *n* = 9 retinas, *p *=* *0.001). Adding AMPA and FMRFamide together resulted in a significant accelerated destaining rate from dark (*p = *0.002), which was not significantly different from that with AMPA alone (1.96 ± 0.25%, *n* = 4, *p = *0.88). Statistical differences were determined with one-way ANOVA (*F*(3,23) = 27.4, *p *=* *8.9 × 10−8). ***E***, ***F***, Time course of FM1-43 destaining in PSAM fish. Adding PSEM^89S^ (30 μm) accelerated destaining (0.83 ± 0.19%, *n* = 9 retinas, *p = *0.044). Adding AMPA accelerated destaining (2.19 ± 0.19%, *n* = 5 retinas, *p *=* *0.0002). Adding PSEM^89S^ and AMPA together resulted in a destaining rate not significantly different from that with AMPA alone (2.05 ± 0.35%, *n* = 5, *p *=* *0.82). Analysis was performed with one-way ANOVA (*F*(3,24) = 14.69, *p *=* *1.2 × 10−5). For ***D*** and ***F*** open circles represent individual data points. Open boxes represent the interquartile range with whiskers. The mean is marked by x and the median by a line.

Previous work (Jackman et al., 2011) suggests that two distinct events initiate negative or positive feedback from HCs to cone photoreceptor terminals, as illustrated in Figure 3*A*. Negative feedback is initiated by a change in HC membrane potential, leading to a change in extracellular proton concentration. This modulates the gating of voltage-gated Ca^2+^ channels in cone terminals, thereby regulating Ca^2+^-dependent exocytosis. Positive feedback is initiated by a local change in intracellular Ca^2+^ in the dendrites of HCs, a process that depends on a change in influx through Ca^2+^-permeable AMPA receptors. The positive feedback signal is still unidentified, although it is known that it too regulates Ca^2+^-dependent exocytosis from photoreceptors. According to this model, altering membrane potential of HCs by activating chemogenetic tools should affect negative feedback, but not positive feedback, which is dependent on Ca^2+^ influx through AMPA receptors.

To test this prediction, we measured the cone terminal FM1-43 unloading rate in darkness, in retinas from both FaNaC (Fig. 3*B*) and PSAM-GlyR fish. In fully dark-adapted FaNaC fish retina with no agonist added, destaining occurred at 0.87 ± 0.08% per minute (*n* = 10 retinas; Fig. 3*C*,*D*). The FM 1-43 destaining rate increased with AMPA (1.89 ± 0.13%, *n* = 9 retinas, *p *<* *1 × 10^−5^ compared with no agonist) and slowed with FMRFamide (0.54 ± 0.07% *n* = 9 retinas, *p *=* *0.001 compared with no agonist) while adding FMRFamide and AMPA together resulted in a destaining rate that was not different from with AMPA alone (1.96 ± 0.25%, *n* = 4, *p = *0.88).

In PSAM-GlyR fish, the destaining rate with no agonists added was 0.41 ± 0.12% per minute (Fig. 3*E*). The slower measured destaining rate as compared with FaNaC retina was attributed to a shorter period of dark adaptation before beginning the imaging session. In the presence of the agonist PSEM^89S^ (30 μm), the destaining rate increased to 0.83 ± 0.19%, significantly faster than the rate in the absence of agonist (*n* = 9 retinas, *p = *0.044; Fig. 3*F*). Addition of 20 μm AMPA significantly increase the FM 1-43 unloading rate compared with dark control (2.19 ± 0.19%, *n* = 5 retinas, *p *=* *0.0002). The addition of both 30 μm PSEM^89S^ and 20 μm AMPA resulted in an acceleration similar to that seen with AMPA alone (2.05 ± 0.35%, *n* = 5, *p *=* *0.82; Fig. 3*F*).

### Chemogenetic manipulation of HCs reduces the b-wave of the ERG

**Figure 4.**
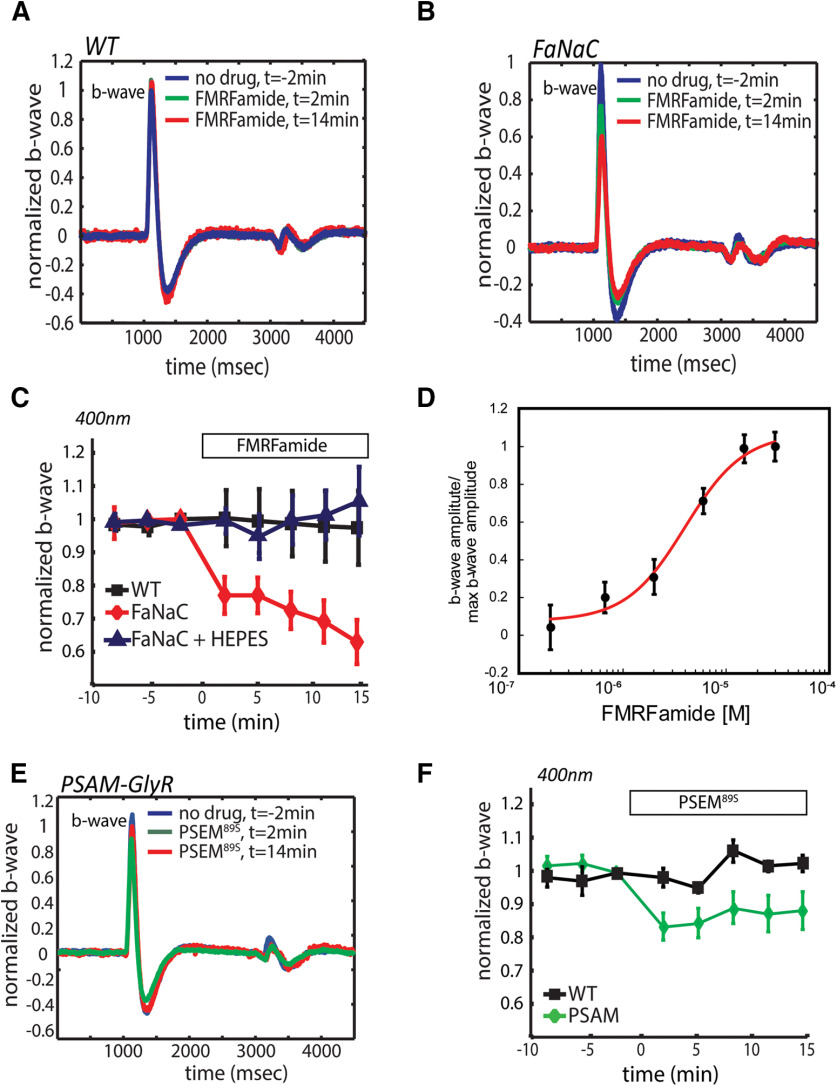
Chemogenetic manipulation of HCs modulates the bipolar cell light response. Application of FMRFamide had no effect on the ERG of WT zebrafish (***A***), but decreased the maximal b-wave amplitude in FaNaC zebrafish (***B***), reaching a maximum ∼40% decrease in the b-wave (***C***). Application of the buffer HEPES (pH 7.35) cancelled the effects of FMRFamide (***C***). FMRFamide response was dose dependent having an EC_50_ of 4.25 μm (***D***). Application of the agonist PSEM^89S^ (30 μm) also caused a decrease in the maximal b-wave amplitude, but to a lesser degree (***E***, ***F***).

Chemogenetic manipulation of HC feedback onto cones should have consequences on the responses of downstream neurons in the visual system, starting with alteration of postsynaptic responses in the outer retina. The ERG b-wave reflects light-elicited synaptic currents, originating primarily in bipolar cells (Stockton and Slaughter, 1989). Following establishment of a stable baseline in control solution, either FMRFamide (10 μm) or PSEM^89S^ (30 μm) was added to the bathing solution. Isolated eyes from WT fish had ERGs that were unaffected by FMRFamide (0.97 ± 0.11, *n* = 10, *p = *0.92; Fig. 4*A*). However, in FaNaC fish, FMRFamide decreased the ERG b-wave (0.63 ± 0.06, *n* = 9, *p = *0.007; Fig. 4*B*,*C*). The effect of FMRFamide on the b-wave was prevented by adding 20 mm HEPES (1.08 ± 0.11, *n* = 7, *p = *0.92), thus supporting the pH mediated effect that HCs have on retinal light responses (Wang et al., 2014; Beckwith-Cohen et al., 2019). The FMRFamide effect was dose dependent with an EC_50_ of 4.25 μm (Fig. 4*D*), consistent with previous estimates of the affinity of FaNaC for the neuropeptide (Schanuel et al., 2008). ERGs from WT fish were also unaffected by PSEM^89S^ (30 μm; *n* = 8, 0.96 ± 0.03, *p = *0.703; Fig. 4*F*). However, in PSAM-GlyR fish, PSEM^89S^ decreased the b-wave (*n* = 7, 0.84 ± 0.05, *p = *0.025; Fig. 4*E*,*F*). This effect was smaller than that of FMRFamide on FaNaC fish, but nonetheless statistically significant. Without agonist, the ERG of PSAM-GlyR fish was indistinguishable from that of WT fish.

### Chemogenetic manipulation of HCs disrupts surround inhibition in RGCs

**Figure 5.**
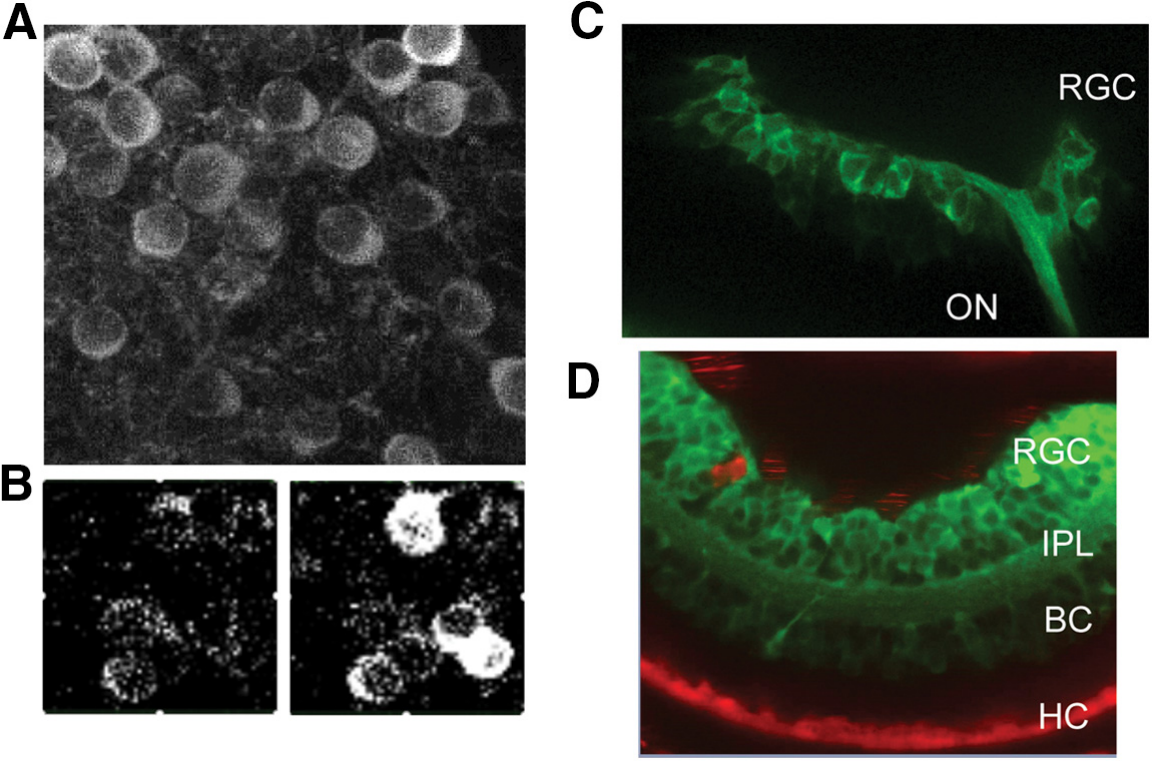
Expression of GCaMP6f in chemogenetic zebrafish lines. ***A***, *Ex vivo* retina of adult HuC-GCaMP6f zebrafish shows eGFP fluorescence in retinal flat mounts using two-photon imaging. ***B***, Light response is easily measured in RGCs before (left) and after (right) a light flash. ***C***, *In vivo* confocal imaging of eGFP in 1 dpf PTU-treated zebrafish larvae of HuC-GCaMP6f and FaNaC crossed transgenic fish. The optic nerve (ON) and the developing retina (RGC) show strong eGFP fluorescence. ***D***, *In vivo* imaging of the same line of fish imaged in ***C*** showing that FaNaC-mCherry is easily visualized in HCs at 2 dpf zebrafish larvae. Some red autofluorescence is generated by scattered pigment cells on the ocular surface. RGC, retinal ganglion cell layer; IPL, inner plexiform layer; BC, bipolar cell layer; HC, horizontal cell layer.

RGCs display an antagonistic center versus surround receptive field, mediated in large part by lateral inhibition in the outer retina. Chemogenetically manipulating the membrane potential of HCs should alter lateral inhibition and therefore change the degree of surround antagonism in RGCs, similar to the effect of pH buffers that disrupt HC signaling (Davenport et al., 2008). To measure RGC responses, we used a transgenic zebrafish expressing the genetically encoded calcium indicator GCaMP6f, under the control of a pan-neuronal promoter (HuC). We observed GCaMP6f expression throughout the nervous system including the brain, spinal cord, and retina (Fig. 5*A–D*). Two-photon Ca^2+^ imaging was used to measure RGC responses to light stimuli (Fig. 5*B*). GCaMP6f fish were crossed with FaNaC or PSAM-GlyR fish to create double transgenic fish lines. In embryos treated with PTU to prevent pigmentation, GCaMP6f could be seen in developing RGCs *in vivo* at 1 dpf (Fig. 5*C*). mCherry, a co-expression marker for FaNaC, could be seen *in vivo* in HCs at 2 dpf (Fig. 5*D*). eGFP, a co-expression marker for PSAM-GlyR, could not be detected concurrently with GCaMP6f because of spectral overlap, although expression was confirmed in intact retinas by immunohistochemistry (Fig. 1*B*) and in dissociated HCs.

**Figure 6.**
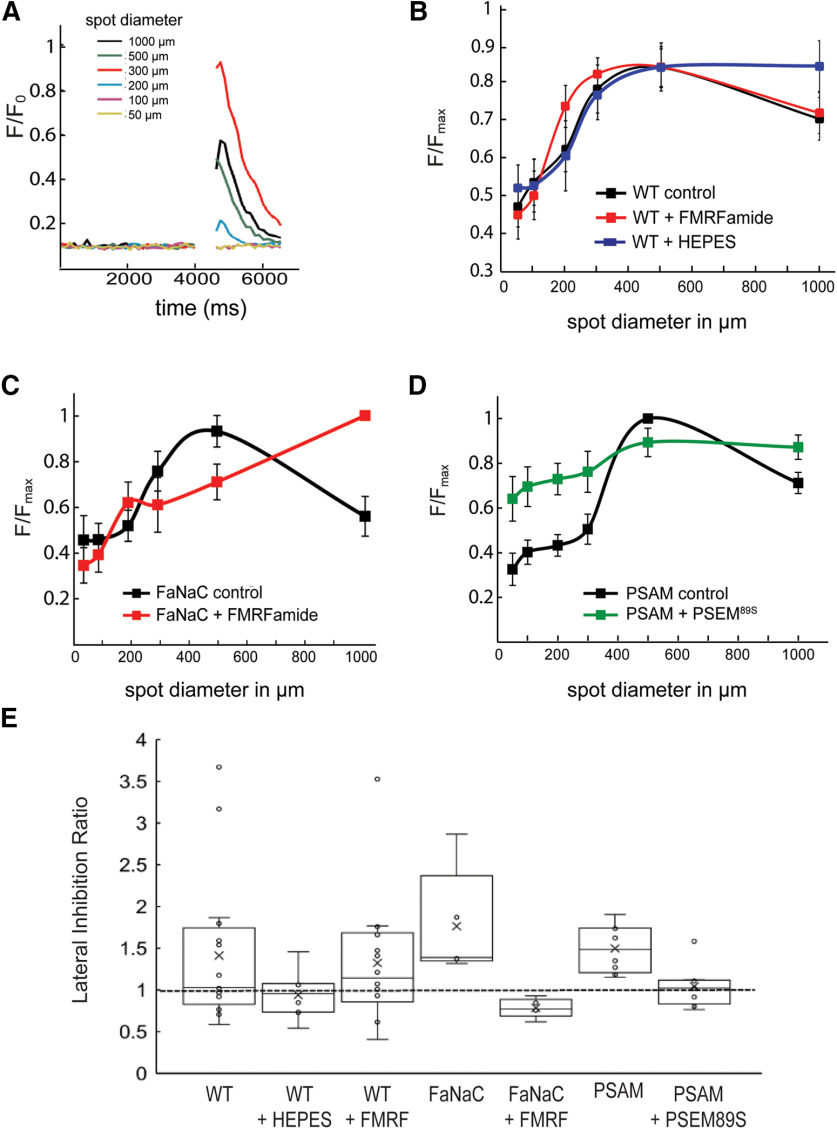
Chemogenetic manipulation of HCs alters lateral inhibition in downstream RGCs. ***A***, Light responses were measured in HuC::GCaMP6f fish with three light stimuli, delivered at six different spot diameters. Calcium transients were recorded using fluorescence imaging. ***B***, WT RGCs responded with maximum change in fluorescence to a 500-μm spot of light, which decreased at 1000 μm, supporting lateral inhibition. This effect was blocked by HEPES, and was unchanged by applying 10 μm FMRFamide. ***C***, Application of FMRFamide on FaNaC retinas perturbed normal RGC center surround response, (***D***) as did application of PSEM^89S^ on PSAM- GlyR retinas. ***E***, The LIR in various conditions shows that application of the cognate agonists to FaNaC and PSAM retina significantly disrupts lateral inhibition. Open circles represent individual data points. Open boxes represent the interquartile range with whiskers. The mean is marked by x and the median by a line.

To measure RGC responses, three repeated light stimuli were delivered at each of six spot diameters (ranging 50–1000 μm) and Ca^2+^ transients were recorded using two-photon imaging (Fig. 6*A*). In WT-GCaMP6f the Ca^2+^ response grew with increasing spot diameter (*n* = 16), but only up to a point. The Ca^2+^ signal reached a maximum at 500 μm (Fig. 6*B*), and then decreased with a larger spot (1000 μm), consistent with surround antagonism (Vessey et al., 2005; Davenport et al., 2008; Klaassen et al., 2011). We calculated the lateral inhibition ratio (LIR), defined as the response at 500 μm/response at 1000 μm. The LIR was 1.41 ± 0.21 for WT. Addition of 20 mm HEPES reduced this effect, resulting in an LIR of 0.95 ± 0.09 (*n* = 8, *p = *0.08). Applying 10 μm FMRFamide had no effect on the LIR of WT fish (1.32 ± 0.19, *n* = 14, *p = *0.76; Fig. 6*B*). FaNaC retinas had surround inhibition before agonist application with an LIR of 1.76 ± 0.26. Application of FMRFamide on FaNaC retinas reduced surround inhibition, resulting in an LIR of 0.78 ± 0.04 (*n* = 4, *p = *0.002; Fig. 6*C*). Likewise, PSAM-GlyR retinas displayed strong surround inhibition before agonist application (LIR = 1.49 ± 0.09), and this surround inhibition was significantly reduced by application of PSEM^89S^ (LIR = 1.04 ± 0.08, *n* = 8, *p = *0.0006; Fig. 6*D*), suggesting that activation of PSAM-GlyR channels that are selectively expressed in HCs also perturbed normal center surround responses of downstream RGCs. A summary of the LIR values in various conditions is shown in Figure 6*E*.

## Discussion

In the dark photoreceptors are depolarized, eliciting a maximal release of glutamate, which acts via a sign conserving synapse on HCs. A light stimulus slows synaptic release thereby hyperpolarizing HCs. Hyperpolarization of HCs in turn activates a negative feedback mechanism that helps restore the rate of transmitter release in the face of continuous light stimulation. Understanding both the mechanisms and the functions of HC feedback to photoreceptors is complicated by the reciprocal nature of the synapse. Chemogenetic tools enable direct manipulation of HC membrane potential, bypassing the photoreceptors to enable functional analysis of feedback. We first examined the effect of HCs on cone vesicular release. Because the Na^+^ equilibrium potential (near +50 mV) is more positive than the HC resting potential (near −40 mV), application of FMRFamide should trigger Na^+^ influx and depolarize cells expressing FaNaC, as we observed in dissociated HCs. This, in turn, should lead to decelerated vesicular release from cones, a prediction that is consistent with our finding that FMRFamide slowed FM1-43 destaining. Our result implies that depolarization of HC alone, through the use of chemogenetics, is sufficient to decrease transmitter release rates from cones. A role for the membrane potential of HCs in the modulation of photoreceptor transmitter release has been demonstrated elsewhere, although the precise underlying mechanism remains elusive (Vessey et al., 2005; Klaassen et al., 2011).

The small increase in FM1-43 release rate that we observed on application of PSEM^89S^ to PSAM-GlyR retina is consistent with a modest hyperpolarization of the HC membrane potential. As PSAM-GlyR is a Cl^–^ channel, hyperpolarization is expected if the equilibrium potential is more negative than the HC resting potential. In agreement with this prediction, we observed consistent hyperpolarizing responses to PSEM^89S^ in dissociated HCs when the normal intracellular Cl^–^ environment was preserved with gramicidin perforated patch recording. We also observed consistently hyperpolarizing responses when we used the specific agonist muscimol to activate GABAA receptors, which are highly Cl^–^ selective. In contrast, application of GABA had complicated effects, in some cases, depolarizing HCs, and in others, generating a biphasic response. This is consistent with GABA activating more than one target. HCs express GABA transporters, which, in addition to shuttling the neurotransmitter, have a ligand-gated Na^+^-selective pore and would thus cause membrane depolarization (Malchow and Ripps, 1990; Cammack and Schwartz, 1993; Dong et al., 1994; Kreitzer et al., 2003; Krause and Schwarz, 2005; Nelson et al., 2008). In fish HCs, blockade of the Cl^–^ pore of GABAA receptors with picrotoxin has a relatively small effect on the GABA response of HCs, suggesting that most of the current is mediated by the transporter (Malchow and Ripps, 1990; Cammack and Schwartz, 1993; Kreitzer et al., 2003; Nelson et al., 2008). Thus, the response to GABA is because of the simultaneous activation of a Na^+^ transport current and a Cl^–^ current, mediated by the GABAA receptor.

To test whether manipulating HC membrane potential altered postsynaptic responses in downstream neurons in the outer retina, we measured responses to light flashes with ERGs. For these experiments we made use of the larval zebrafish (<8 dpf) which has several advantages compared with adult fish. First, a surface electrode on the cornea is sufficient to detect responses in the larvae, while measurements in the adult require perforation of the eye. Moreover, pharmacological agents can easily penetrate larval zebrafish. Thus, FMRFamide and PSEM^89S^ can be applied and light responses can be recorded without interfering with ocular integrity. Although the ERG of zebrafish larvae does not fully mature until 21–24 dpf, a strong b-wave response to ultraviolet light occurs at 4–5 dpf (Saszik et al., 1999). The minimal a-wave seen in our ERGs (Fig. 3*A*,*B*,*E*) is typical of the cone-dominant retina of larval zebrafish, which have yet to develop rods (Seeliger et al., 2002).

Application of either FMRFamide or PSEM^89S^ in the appropriate transgenic zebrafish line, decreased the ERG b-wave, which is thought to reflect synaptic responses primarily from ON-bipolar cells. In principle, there are several ways that HCs might contribute to the b-wave of the ERG. First, synaptic currents in the HCs themselves might add to the trans-retinal electrical field in a way that could be detected in the ERG recording, contributing in a direct manner. This seems unlikely, as selective block of ON-bipolar cell light responses with L-AP4 eliminates the b-wave (Stockton and Slaughter, 1989; Thoreson and Miller, 1994; Robson and Frishman, 1995; Saszik et al., 2002). Second, feed-forward synapses from HCs to bipolar cells might also generate a synaptic current that directly contributes to the b-wave. While such a feed-forward connection has long been sought, evidence for a substantial synaptic response in bipolar cells that can be attributed to HCs remains scant, but cannot be ruled out (Thoreson and Mangel, 2012). Finally, feedback from HCs onto rod and cone photoreceptors will modulate their neurotransmitter release and alter responses of bipolar cells in an indirect manner. Because of the signal amplification of the photoreceptor to bipolar cell synapse (Ashmore and Copenhagen, 1980), HC feedback onto cone terminals might have a larger effect on the b-wave amplitude than feedforward input onto ON-bipolar cells. Thus, it is likely that the decrease in b- wave amplitude observed here results from HC-mediated modulation of synaptic transmission from cones to ON-bipolar cells.

Our data demonstrate that FMRFamide decelerates release while PSEM^89S^ accelerates release from cones, and yet both agonists decreased the amplitude of the b-wave, a seemingly counterintuitive result. However, this finding is consistent with our understanding of the mechanism of modulation of release by HC membrane potential: The relationship between photoreceptor terminal voltage and I_Ca2+_ activation can be expressed using a Boltzmann function, with the steepest portion of the curve ideally located at the dark potential of the photoreceptor, such that small hyperpolarizations of the cone terminal dramatically decrease Ca^2+^ channel activation and therefore reduce transmitter release. Hyperpolarization of HC membrane potential with extrinsic current or light annuli shifts I_Ca2+_ such that activation increases at more negative photoreceptor voltages, thereby contributing to restoration of transmitter release from photoreceptors during maintained illumination (Verweij et al., 1996; Hirasawa and Kaneko, 2003; Cadetti and Thoreson, 2006; Thoreson et al., 2008). Chemogenetic manipulation of HC membrane potential in either direction would be expected to shift the cone Ca^2+^ activation curve away from the steepest portion of the relationship, resulting in smaller changes in transmitter release in response to light-evoked hyperpolarization of cone membrane potential. The decrease in b-wave amplitude that we observe here supports this hypothesis.

This finding is consistent with the idea that the set point of retinal HCs, in the absence of chemogenetic perturbation, is situated to produce the maximal effect of light on cone transmitter release.

The effects of chemogenetic manipulation are consistent with perturbation of negative feedback, which is dependent exclusively on changes in HC membrane potential (Warren et al., 2016) and inconsistent with effects on positive feedback, which are triggered by influx of Ca^2+^ through Ca^2+^-permeant AMPA receptors (Jackman et al., 2011). In the presence of AMPA stimulation, chemogenetic modulation of HC voltage was ineffective at altering transmitter release rates (Fig. 3); consistent with the idea that changing HC membrane potential would have only modest effects on the driving force for Ca^2+^ influx through Ca^2+^-permeant AMPA receptors. Thus, positive and negative feedback appear to be regulated through entirely separable and independent mechanisms.

In addition to reducing the b-wave, application of either FMRFamide or PSEM^89S^ to retinas expressing their cognate receptors flattened the center surround response curve of downstream RGCs, and decreased the LIR. These chemogenetic interventions affected the center surround response in a similar manner to that seen when protons are buffered with HEPES (Davenport et al., 2008; Crook et al., 2009). This is consistent with the idea that uniform biasing of HC membrane potential across the retina with FMRFamide and PSEM^89S^ removes local differences in inhibitory feedback at the border of the light stimulus that are essential for generating surrounds in the inner retina.

For these experiments, we measured the response of RGCs before and after drug application; however, we did not classify the RGC type of each recorded cell. Using morphologic and functional experiments there are estimated 36 or more RGC types in the retina, and this is in addition to displaced amacrine cells present within the ganglion cell layer (Baden et al., 2016). Therefore, one could anticipate that chemogenetic perturbation of HCs may elicit a different response effect on different types of RGCs. This is further supported by experiments showing that perturbing HCs using PSEM^89S^ in retinas with HC-expressed PSAM-GlyR has different effects on the kinetics of different RGC types (Drinnenberg et al., 2018). Interpretation of these results is further complicated by the understanding that there are three known types of HCs (Connaughton et al., 2004), which were targeted without discrimination. Development of zebrafish lines in which either FaNaC or PSAM-GlyR is selectively expressed in specific HC subtypes would provide a powerful tool for dissecting the relative importance of each HC for generation of antagonistic surrounds in downstream RGCs.

In summary, we show a method that allows direct perturbation of HC cell membrane potential to investigate the effects that HCs have on complex retinal responses to light. While perturbing HC membrane potential cannot overcome the Ca^2+^ drive for cone synaptic release, shifting of the membrane potential by depolarizing or hyperpolarizing it greatly decreases the downstream retinal responses to a light stimulus. The use of the chemogenetic tools we present, with or without targeting to specific HC types, could therefore be further used to explore the direct effects that HCs have on specific types of ganglion cells, and on visual function, including color and contrast sensitivity.

## Synthesis

Reviewing Editor: Juan Burrone, King's College London Decisions are customarily a result of the Reviewing Editor and the peer reviewers coming together and discussing their recommendations until a consensus is reached. When revisions are invited, a fact-based synthesis statement explaining their decision and outlining what is needed to prepare a revision will be listed below. The following reviewer(s) agreed to reveal their identity: Wallace Thoreson.

The authors use FaNaC Na+ and PSAM-GlyR Cl- channel chemogenetic transgenic zebrafish to modulate horizontal cell membrane potential and assay the effects on cone transmitter release with FM1-43 destaining, the b-wave of the ERG, and the inhibitory surrounds of ganglion cells. The work provides a unique description of how HCs impact on the visual information processing pathways and makes use of various techniques to assess this. Although the findings and the approach were found to be innovative and informative, there remain areas of concern that need to be addressed by the authors. Below we provide a list of these concerns.

1. It is highly surprising that PSEM89S activation of the PSAM-GlyR chloride channel in isolated horizontal cells produced hyperpolarizations at Vrest (-40 to -45 mV). Although the 30 μM PSEM89S was used in flat-mounted, whole retina prep, 500 μM PSEM89S was applied to isolated cells. The perforated patch-clamp recordings need to be repeated with (less than or equal to) 30 μM PSEM89S. This is an important issue that needs to be addressed.

2. In different species (zebrafish, catfish, salamander, axolotl, mudpuppy, stingray, rabbit, mouse) where horizontal cell ECl was determined and/or GABAA receptors activated by GABA/muscimol, ECl was found to be around -20 to -30 mV and GABA/muscimol produced depolarizations at Vrest, respectively. Grove et al. (2019) Figure 5A-C showed a gramicidin perforated-patch recording of a mouse horizontal cell and the summary I-V relation of the chloride currents evoked by muscimol. Erev for Cl- was estimated to be -28mV under physiological conditions. In a Grove et al. (2019) supplemental figure, PSEM89S was applied to a mouse PSAM-GlyR-expressing horizontal cell. 10 μM PSEM89S evoked a current that reversed around ECl, as expected for a modified GlyR Cl- channel. Nelson et al. (2008) report GABA produced depolarizations at Vrest in both morphologically identifiable types of isolated zebrafish horizontal cells. What do the authors think is different about their PSAM-GlyR transgenic zebrafish that PSEM89S produces hyperpolarizations?

3. It may also be worth assessing changes in membrane resistance, which will also take place during activation of PSAM-GlyR channels. Changes in Rm would also have important consequences on the excitability of HCs.

4. Although the authors now provide data in isolated HCs for the effect of PSEM89S, there are no figures showing the effect of FMRFamide on membrane potential, only mentions in the text. It would be important to show this data set too. In addition, there are no statistics on any of the measures provided in Fig. 1C and D.

5. The methods section is very sparse and confusing. This section needs more work. For example:

The constitution of the external bath solution for both the patch-clamp and the FM1-43 destaining experiments are not stated.

The specifics of the imaging setup (microscope objectives, filters sets, etc) should be listed.

The section of FM1-43 imaging is unclear. How was FM1-43 measured? Did the authors use 2-photon microscopy (what laser wavelength, what filters?) or an Argon laser at 488 nm? Both are mentioned in the methods. In either case, did the laser stimulate the photoreceptors and cause release? Are there controls to account for this?

6. The FM1-43 destaining takes place over long periods (minutes). Is there a negative control (eg - absence of extracellular calcium) to account for non-specific loss of signal?

7. I suggest the authors consider highlighting in the abstract the differences in effects of FMRFamide and AMPA which showed that positive and negative feedback are separable and distinct. In addition to the technical demonstration of genetic manipulations, I think this is perhaps the most significant result of this study.
